# The role of [^18^F]F-DOPA PET/CT in diagnostic and prognostic assessment of medullary thyroid cancer: a 15-year experience with 109 patients

**DOI:** 10.1530/ETJ-24-0089

**Published:** 2024-07-13

**Authors:** Zhaoqi Zhang, Josef Yu, Eva Rainer, Lindsay Hargitai, Zewen Jiang, Georgios Karanikas, Tatjana Traub-Weidinger, Richard Crevenna, Marcus Hacker, Shuren Li

**Affiliations:** 1 Department of Biomedical Imaging and Image-guided Therapy, Division of Nuclear Medicine, Medical University of Vienna, Vienna, Austria; 2 Department of Nuclear Medicine, The Fourth hospital of Hebei Medical University, Shijiazhuang, Hebei, China; 3 Department of Biomedical Imaging and Image-guided Therapy, Division of General and Pediatric Radiology, Medical University of Vienna, Vienna, Austria; 4 Department of General Surgery, Medical University of Vienna, Vienna, Austria; 5 Department of Physical Medicine, Rehabilitation and Occupational Medicine, Medical University of Vienna, Vienna, Austria.

**Keywords:** calcitonin, DOPA, medullary thyroid cancer (MTC), metabolic tumour volume (MTV), PET/CT, prognosis

## Abstract

**Objective:**

Correct diagnosis and prognostic evaluation of medullary thyroid cancer (MTC) are crucial to treat patients. The purpose of this study was to evaluate the diagnostic and prognostic value of [^18^F]F-DOPA PET/CT in patients with MTC.

**Methods:**

We reviewed MTC patients who underwent [^18^F]F-DOPA PET/CT from June 2008 to November 2023. Clinical characteristics, follow-up data, and the following [^18^F]F-DOPA PET/CT parameters were recorded: maximum standardized uptake value (SUV_max_), mean standardized uptake value (SUV_mean_), metabolic tumor volume (MTV), and SUV_mean_ of multiple organs. The diagnostic value of PET/CT for the detection of tumor lesions was calculated. Serum basal calcitonin (bCt) and stimulated calcitonin (sCt) were determined. Receiver operating characteristics, Kaplan–Meier, and Cox regression analyses were performed.

**Results:**

In total, 109 patients (50 women, 59 men; average age, 55 ± 14 years) were included in the analysis. The patient-related sensitivity, specificity, and accuracy of [^18^F]F-DOPA PET/CT were 95%, 93%, and 94%, respectively. The lesion-related sensitivity, specificity, and accuracy were 65%, 99%, and 72%, respectively. The optimal cutoff values of bCt, sCt, and CEA to obtain positive [^18^F]F-DOPA PET/CT results were 64 pg/mL, 1808 pg/mL, and 4 µg/L, respectively. Patients with negative [^18^F]F-DOPA PET/CT had longer overall survival than patients with positive [^18^F]F-DOPA PET/CT results (*P* = 0.017). Significant positive correlations were found between bCt, sCt, and CEA with SUV_max_, SUV_mean_, and MTV of [^18^F]F-DOPA PET/CT (*P* < 0.001). [^18^F]F-DOPA PET/CT results and MTV may be useful for the evaluation of the prognosis of patients with recurrent MTC, while age and MTV were independent prognostic factors in patients with primary MTC. For all patients, SUV_mean_ of the left kidney, liver, aorta, and pancreas might be used to independently predict OS.

**Conclusion:**

[^18^F]F-DOPA PET/CT had great value for diagnosis and prognostic assessment in patients with MTC. The DOPA PET/CT parameter SUV_mean_ and MTV showed significant association with OS.

## Introduction

Medullary thyroid cancer (MTC) accounts for about 2% of all thyroid cancers. About 20–25% of MTC cases are hereditary, caused by a mutation in the RET proto-oncogene. However, in most cases, MTC is sporadic with no underlying germline mutation (1, 2).

Patients with MTC have a high risk of developing distant metastases. Up to 23% of patients present with lymph nodes or distant metastases at the time of diagnosis, which is the main reason for MTC-related death. Therefore, early diagnosis of the primary tumor and metastases is essential for outcomes. Basal calcitonin (bCt) and carcinoembryonic antigen (CEA) can be used for diagnosis and follow-up of MTC. In case of increased bCt levels, a calcium stimulation test may be performed to determine stimulated calcitonin (sCt) levels, which may enhance accuracy in the diagnosis of MTC (3, 4).

Correct identification of primary and/or metastatic/recurrent MTC (RMTC) by non-invasive methods is critical for diagnosis, therapy, and prognosis. Determination of basal calcitonin (bCt) and stimulated calcitonin (sCt), as well as ultrasound of the neck and CT and MRI, are valuable for diagnosis. However, their ability to provide metabolic information is limited (5). [^18^F]FDG PET/CT can provide both anatomical and metabolic information; however, it has been shown to have low sensitivity for the detection of MTC (5, 6, 7). [^18^F]F-DOPA PET/CT has been demonstrated to be useful for the detection of PMTC and RMTC (8, 9, 10, 11, 12). However, only a limited number of patients were included in most previous studies of [^18^F]F-DOPA PET/CT. The optimal cutoff values of bCt, sCt, and CEA for positive [^18^F]F-DOPA PET/CT results are still controversial. Furthermore, the prognostic value of [^18^F]F-DOPA PET/CT and [^18^F]F-DOPA PET/CT parameters in patients with MTC are still unknown.

In this study, we investigated the usefulness of [^18^F]F-DOPA PET/CT in 109 patients with MTC for the following purposes: (1) the diagnostic value of [^18^F]F-DOPA PET/CT in patients with MTC; (2) the prognostic value of [^18^F]F-DOPA PET/CT in patients with MTC; (3) the correlation of Ct and CEA levels with [^18^F]F-DOPA PET/CT results and exploration of the best thresholds of Ct and CEA for imaging MTC with [^18^F]F-DOPA PET/CT; and finally (4) the prognostic value of [^18^F]F-DOPA PET/CT parameters in patients with MTC.

## Materials and methods

### Patients

This study was approved by the Ethics Committee of the Medical University of Vienna. We retrospectively reviewed data from all the patients with MTC who underwent [^18^F]F-DOPA PET/CT at the Department of Biomedical Imaging and Image-guided Therapy of Medical University of Vienna from June 2008 to November 2023. The inclusion criteria were as follows: (1) age ≥ 18 years; (2) primary MTC (PMTC) before surgery or suspected recurrent or persistent disease after initial surgery, indicated by elevated calcitonin levels (> 15 pg/mL in PMTC and > 1 pg/mL in suspected RMTC), or confirmed by cytology, histopathological exam, or other imaging tests during follow-up; (3) complete clinical and imaging data. The exclusion criteria were those which did not meet the inclusion criteria. The 8th edition of the International Union against Cancer (UICC) staging system (13) for medullary thyroid cancer was used for stage classification.

### bCt, sCt, and CEA levels

The Ct concentrations were determined using an immunochemiluminescence assay (ICMA) from Diagnostic Products Corporation (DPC, Los Angeles, CA, USA), as described previously (3). The ICMA results of this study were comparable with findings in other studies due to the worldwide use in many publications (14, 15).

Blood samples for sCt determination during the calcium-stimulated test were performed according to our previous publication described in detail (3).

The CEA levels were measured with a commercially available electrochemiluminescence immunoassay kit (Roche Diagnostics).

### [^18^F]F-DOPA PET/CT imaging

All [^18^F]F-DOPA PET/CT examinations were performed from the skull base to the upper thighs using a 64-row multidetector hybrid system (Biograph TruePoint 64; Siemens) with an axial field-of-view of 216 mm, a PET sensitivity of 7.6 cps/kBq, and a transaxial PET resolution of 4–5 mm (full-width at half-maximum). All PET/CT examinations were performed 60 min after intravenous administration of 3 MBq/kg body weight [^18^F]F-DOPA, with 4 min/bed position, four iterations per 21 subsets, a 5 mm slice thickness, and a 168 × 168 matrix, using the TrueX algorithm based on the point-spread function.

### PET/CT data analysis

The [^18^F]F-DOPA PET/CT examinations were evaluated by two experts in this field. Thyroid nodules, lymph nodes, and distant metastasis with visually higher [^18^F]F-DOPA uptake than the surrounding background activity were defined as DOPA-positive lesions, while those with equal or lower [^18^F]F-DOPA uptake than the surrounding background were defined as [^18^F]F-DOPA-negative lesions. A lesion was considered true-positive if histopathology was positive or if it showed progression at follow-up examinations. A lesion was considered true-negative if histopathology was negative or if follow-up examinations did not show any pathological morphological result for at least 18 months, and the Ct level was negative in at least two determinations. A lesion was considered false-positive if the [^18^F]F-DOPA scan was positive and if histopathology was negative or if it showed no progression at follow-up examinations. A lesion was considered as false-negative if the [^18^F]F-DOPA scan was negative and if histopathology was positive or if follow-up examinations showed growth of the lesion(s).

SUV_max_, SUV_mean_, and MTV of lesions on [^18^F]F-DOPA PET/CT images were obtained using 3D slicer software. Multiple-organ objective segmentation (MOOSE) software was used to generate SUV_mean_ values of multiple organs.

### Follow-up

Follow-up was performed after [^18^F]F-DOPA PET/CT scan. Overall survival (OS) was defined as the time interval from the date of PET/CT imaging to death related to MTC or the date of the last follow-up. OS was chosen as an endpoint to estimate the prognostic value of clinical data and PET/CT parameters. Given the longer survival time of MTC patients, 120 months (10 years) survival time of patients were compared and analyzed in this study.

### Statistical analysis

Correlation and cut-off values were determined using Spearman's correlation, the Chi-square test, Fisher’s exact test, and receiver operating characteristic (ROC) curves. For all statistical tests, values of *P* <0.05 were considered statistically significant. Kaplan–Meier’s method was applied to generate survival curves, and the log-rank test was used for comparison. Univariate and multivariate logistic regression were used to determine the relationship between various relevant variable and [^18^F]F-DOPA PET/CT results. Cox regression was used to determine the relationship between various relevant variable and OS. Statistical analyses were performed using IBM SPSS Statistics version 25.0 (IBM). MedCalc version 22.016 (MedCalc Software Ltd, Belgium) was used to compare the differences between ROCs. R language (version 4.3.2) was used to generate a heatmap of the correlation among different organs and a heatmap of the expression correlation of bCt, age, OS, and SUV_mean_ of multiple organs.

## Results

The study population comprised 109 patients (50 females, 59 males, mean age 55 ± 14 years, range 20–84 years). Fifty patients had PMTC and 59 patients RMTC. Twelve patients presented with hereditary MTC (HMTC; multiple endocrine neoplasia 2 syndrome (MEN2)-associated MTC), 97 patients with sporadic MTC (SMTC). Eighty-one patients were confirmed as true positives (18 in stage I, eight in stage II, seven in stage III, 30 in stage IVa, and 18 in stage IVc according to the criteria of the 8th edition of the UICC staging system (13)), while the remaining 28 were true negatives.

## Patient-related diagnostic efficiency

In this study, [^18^F]F-DOPA PET/CT scans revealed positive results in 79 patients (41 females, 38 males), with two false positives and four false negatives. This yielded a patient-related sensitivity of 95% (77/81), specificity of 93% (26/28), and accuracy of 94% (103/109) (Table 1).

**Table 1:** Diagnostic efficiency of [^18^F]F-DOPA PET.

	TP	TN	Sensitivity	Specificity	Accuracy	PPV	NPV
Patient-related diagnostic efficiency							
Total	81	28	95% (77/81)	93% (26/28)	94% (103/109)	97% (77/79)	87% (26/30)
F	41	9	98% (40/41)	89% (8/9)	96% (48/50)	98% (40/41)	89% (8/9)
M	40	19	93% (37/40)	95% (18/19)	93% (55/59)	97% (37/38)	86% (18/21)
*P* ( F vs M)			0.590	0.548	0.832	1.000	1.000
PMTC	50	0	92% (46/50)		92% (46/50)	100% (46/46)	
RMTC	31	28	100% (31/31)	93% (26/28)	97% (57/59)	94% (31/33)	100% (26/26)
*P* (PMTC vs RMTC)			0.277	–	0.529	0.171	–
HMTC	7	5	100% (7/7)	100% (5/5)	100% (12/12)	100% (7/7)	100% (5/5)
SMTC	74	23	95% (70/74)	91% (21/23)	94% (91/97)	97% (70/72)	84% (21/25)
*P* (HMTC vs SMTC)			1.000	1 000	1 000	1.000	1.000
Lesion-related diagnostic efficiency							
Total	775	205	65 % (501/775)	99% (203/205)	72% (704/980)	99% (501/503)	43% (203/477)
F	300	93	60% (181/300)	99% (92/93)	69% (273/393)	99% (181/182)	44% (92/211)
M	475	112	68% (321/475)	99% (111/112)	73% (431/587)	99% (320/321)	42% (111/266)
*P* ( F vs M)			0.040	1.000	0.177	1.000	0.681
PMTC	563	0	51% (289/563)		51% (289/563)	100% (289/289)	
RMTC	212	205	100% (212/212)	99% (203/205)	99% (415/417)	99% (212/214)	100% (203/203)
*P* (PMTC vs RMTC)			0.000		0.000	0.181	
HMTC	51	26	25% (13/51)	100% (26/26)	51% (39/77)	100% (13/13)	41% (26/64)
SMTC	724	179	67% (488/724)	99% (177/179)	74% (665/903)	99% (488/490)	43% (177/413)
*P* (HMTC vs SMTC)			0.000	1.000	0.000	1.000	0.737
LN	510	58	54% (276/510)	98% (57/58)	59% (333/568)	99% (276/277)	20% (57/291)
DM	185	91	88% (162/185)	100% (91/91)	92% (253/276)	100% (162/162)	80% (91/114)

DM, distant metastases; F, female; HMTC, hereditary MTC; LK, lymph node lesions; M, male; NPV, negative predictive value; PMTC, primary MTC; PPV, positive predictive value; RMTC, recurrent MTC; SMTC, sporadic MTC; TN, true negative; TP, true positive.

[^18^F]F-DOPA PET/CT scans were positive in 92% (46/50) of patients with PMTC and in 100% (31/31) of those with RMTC (Table 1). In patients with HMTC, 100% (7/7) had positive scans, compared to 95% (70/74) of those with SMTC (Table 1). Regarding gender-specific results, 40 female patients showed positive [^18^F]F-DOPA PET/CT findings (one false positive and one false negative), with a sensitivity of 98% (40/41), specificity of 89% (8/9), and accuracy of 96% (48/50). In contrast, 37 male patients had positive scans (one false positive and three false negatives), yielding a sensitivity of 93% (37/40), specificity of 95% (18/19), and accuracy of 93% (55/59). Statistical analysis indicated no significant gender differences in sensitivity (*P* = 0.590), specificity (*P* = 0.548), and accuracy (*P* = 0.832).

## Lesion-related diagnostic efficiency

Of all 109 patients with MTC, in total, 980 lesions were detected, including 775 true positive and 205 true negative lesions. Among these, 503 lesions were [^18^F]F-DOPA positive (including two false positives), and 477 lesions were [^18^F]F-DOPA negative (including 274 false negatives), resulting in a lesion-related sensitivity of 65% (501/775), specificity of 99% (203/205), and accuracy of 72% (704/980) (Table 1) .

Furthermore, lymph node metastases were evaluated in 568 lesions, comprising 510 positive and 58 negative lesions. [^18^F]F-DOPA PET/CT detected 277 positive lesions (including one false positive) and 291 negative lesions (with 234 false negatives), yielding a sensitivity of 54% (276/510), specificity of 98% (57/58), and an accuracy of 59% (333/568) for lymph node metastases.

Moreover, among the 276 lesions identified as distant metastases (185 were true positives and 91 true negatives), [^18^F]F-DOPA imaging detected 162 as positive and 114 as negative (including 23 false negatives). This demonstrated a sensitivity of 88% (162/185), a specificity of 100% (91/91), and an overall accuracy of 92% (253/276) for detecting distant metastases.

[^18^F]F-DOPA false negative lesions were mainly observed in pulmonary nodules and cervical lymph nodes smaller than 1 cm. The detail lesion-related diagnostic values of [^18^F]F-DOPA PET/CT in females and males, PMTC and RMTC, HMTC and SMTC, are shown in Table 1.

## Cut-off values for imaging with [^18^F]F-DOPA PET/CT

As shown in Table 2, optimal cut-off values for positive [^18^F]F-DOPA PET/CT scans were found at bCt levels ≥ 64 pg/mL with a sensitivity of 89%, a specificity of 73%, and an AUC of 0.848, as well as at sCt levels ≥ 1808 pg/mL with a sensitivity of 79%, a specificity of 95%, and an AUC of 0.808.

**Table 2:** Cut-off values for imaging with [^18^F]F-DOPA PET/CT.

	bCt (pg/mL)			sCt (pg/mL)			CEA (µg/L)		
	Values	Sensitivity	Specificity	Values	Sensitivity	Specificity	Values	Sensitivity	Specificity
Total	≥ 64	89%	73%	≥ 1808	79%	95%	≥ 4	86%	78%
Female	≥ 64	90%	89%	≥ 767	89%	100%	≥ 5	86%	71%
Male	≥ 264	63%	95%	≥ 2322	76%	100%	≥ 4	93%	82%
PMTC	≥ 63	80%	100%	≥ 2241	75%	100%	≥ 3	95%	75%
RMTC	≥ 156	98%	95%	≥ 2498	77%	100%	≥ 5	91%	79%
F-RMTC	≥ 84	100%	88%	≥ 955	88%	100%	≥ 5	100%	83%
M-RMTC	≥ 158	81%	83%	≥ 4483	78%	100%	≥ 10	73%	100%
HMTC	–	–	–	–	–	–	–	–	–
SMTC	≥ 64	90%	72%	≥ 2241	79%	100%	≥ 10	70%	93%

bCt, basal calcitonin; CEA, carcinoembryonic antigen; F-RMTE, female patients with RMTC; HMTC, hereditary MTC; M-RMTC, male patients with RMTC; PMTC, primary MTC; RMTC, recurrent MTC; SMTC, sporadic MTC; sCt, stimulated calcitonin; –, not determined.

Furthermore, the optimal cut-off values for imaging in male patients were ≥ 264 pg/mL for bCt with a sensitivity of 63 % and a specificity of 95%, and over 2322 pg/mL for sCt with a sensitivity of 76% and a specificity of 100%. The best cut-off values for imaging with [^18^F]F-DOPA PET/CT in female patients were ≥64 pg/mL for bCt with a sensitivity of 90% and a specificity of 89%, and ≥767 pg/mL for sCt with a sensitivity of 89%, and a specificity of 100%. Although the optimal cut-off values in male were higher than in female patients, there were no significant differences in both bCt (*z* = 0.002, *P* = 0.999) and sCt (*z* = 0.806, *P* = 0.420) ROCs between female and male patients.

The best cut-off value for CEA was 4 µg/L with a sensitivity of 86%, a specificity of 78%, and an AUC of 0.859. Furthermore, the optimal cut-off value for CEA in female patients was 5 µg/L with a sensitivity of 86%, a specificity of 71%, and an AUC of 0.739, while in male patients it was 4 µg/L with a sensitivity of 93%, a specificity of 82%, and an AUC of 0.915. There were no significant differences in CEA ROCs between female and male patients (*z* = 1.345, *P* = 0.179).

The optimal cut-off values for imaging in PMTC and RMTC patients, female patients with RMTC, male patients with RMTC, and SMTC patients are shown in Table 2.

## Correlations of calcitonin and CEA levels with [^18^F]F-DOPA uptake

The mean bCt, sCt, and CEA levels of patients with positive [^18^F]F-DOPA PET/CT results were 2946 ± 5863 pg/mL, 31,565 ± 54,679 pg/mL, and 198 ± 957 µg/L, while in patients with negative [^18^F]F-DOPA PET/CT results were 115 ± 338 pg/mL, 754 ± 678 pg/mL, and 65 ± 263 µg/L (*t =* 4.273, 4.101 and 0.579, *P* < 0.001, *P* < 0.001 and *P* = 0.564).

Significant positive correlations were observed between bCt and [^18^F]F-DOPA uptake (*r* = 0.621, 0.566, and 0.705, all *P* < 0.001) as well as between sCt and [^18^F]F-DOPA uptake (*r* = 0.552, 0.494 and 0.729, all *P* < 0.001) measured by SUV_max_, SUV_mean_, and MTV. Significant positive correlations were also shown between CEA and SUV_max_, SUV_mean_, and MTV (*r* = 0.608, 0.577, and 0.729, all *P* < 0.001).

In the female cohort, statistically significant positive correlations were observed between bCt and SUV_max_, SUV_mean_, and MTV (*r* = 0.610, 0.555, and 0.692, respectively; all *P* < 0.001). Similar positive correlations were also found between sCt and SUV_max_, SUV_mean_, and MTV (*r* = 0.363, 0.371, and 0.599, respectively; all *P* < 0.05). Additionally, in female patients, CEA levels also showed significant positive correlations with SUV_max_, SUV_mean_, and MTV (*r* = 0.475, 0.410, and 0.535, respectively; all *P* < 0.05).

In the male cohort, bCt exhibited significant positive correlations with SUV_max_, SUV_mean_, and MTV (*r* = 0.598, 0.532, and 0.697, respectively; all *P* < 0.001), as did sCt (*r* = 0.649, 0.585, and 0.799, respectively; all *P* < 0.001). Furthermore, CEA levels in male patients were positively correlated with SUV_max_, SUV_mean_, and MTV (*r* = 0.709, 0.695, and 0.864, respectively; all *P* < 0.001).

## Univariate and multivariate logistic regression analysis of [^18^F]F-DOPA PET/CT

In the univariate analysis, several factors were found to be significantly correlated with the results of [^18^F]F-DOPA PET/CT imaging. These included gender, stage, PMTC and RMTC, and bCt, as shown in Table 3. In the multivariate analysis, only stage, PMTC and RMTC, and bCt showed their significant correlation with the PET/CT results. Other variables examined did not demonstrate predictive value for the PET/CT outcomes.

**Table 3:** Univariate and multivariate logistic regression analysis of PET results on the variables tested

Variable	Univariate analysis		Multivariate analysis	
	HR (95% CI)	*P*	HR (95% CI)	*P*
Age	1.011 (0.981–1.041)	0.484	–	–
Gender	0.397 (0.162–0.974)	0.044	0.813 (0.166–3.990)	0.799
Stage	5.037 (2.571–9.868)	0.000	3.533 (1.864–6.697)	0.000
SMTC and HMTC	2.057 (0.599–7.070)	0.252	–	–
PMTC and RMTC	9.061 (2.887–28.435)	0.000	21.549 (3.740–124.165)	0.001
bCt	1.003 (1.001–1.005)	0.006	1.002 (1.000–1.004)	0.020

## Follow-up and OS analysis of MTC

**Figure 1:**
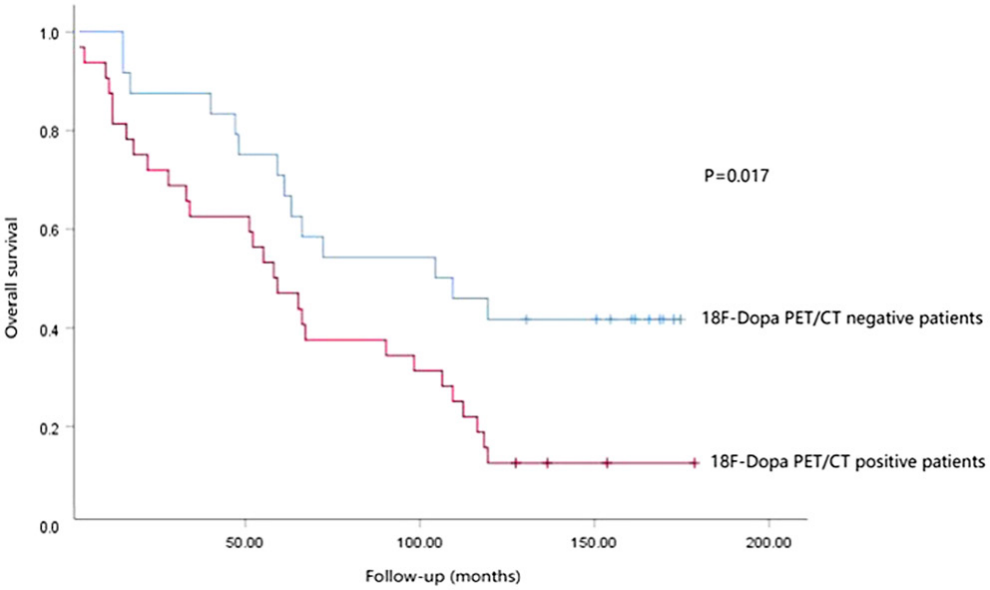
Overall survival (OS) difference between [^18^F]F-DOPA PET-positive and negative patients with recurrent MTC (RMTC).

In the cohort of RMTC patients, survival analysis was conducted on 95% (56 out of 59) of the patients, as three were lost to follow-up. The analysis revealed that patients with negative [^18^F]F-DOPA PET/CT scans had a significantly longer mean survival time of 107 ± 13 months, compared to 70 ± 10 months for those with positive scans (*χ*² = 5.733, *P* = 0.017) (Fig. 1). Univariate Cox regression analysis identified PET/CT results, MTV, and bCt as significant factors correlated with OS (Table 4). However, multivariate analysis confirmed only PET/CT results and MTV as predictive of OS; other variables showed no predictive value.

**Table 4:** Univariate and multivariate Cox regression analysis of OS on the variables tested.

Variable	RMTC patients				PMTC patients			
	Univariate analysis		Multivariate analysis		Univariate analysis		Multivariate analysis	
	HR (95% CI)	*P*	HR (95% CI)	*P*	HR (95% CI)	*P*	HR (95% CI)	*P*
PET/CT results	2.154 (1.129–4.110)	0.020	3.006 (1.010–8.948)	0.048	0.999 (0.307–3.253)	0.998	–	–
MTV	1.012 (1.005–1.018)	0.001	1.016 (1.002–1.030)	0.021	1.002 (1.001–1.003)	0.007	1.002 (1.000–1.004)	0.041
SUV_max_	1.032 (0.996–1.069)	0.086	0.984 (0.908–1.066)	0.686	0.998 (0.949–1.050)	0.947	–	–
SUV_mean_	1.137 (0.978–1.321)	0.094	0.863 (0.596–1.249)	0.434	1.119 (0.887–1.412)	0.343	–	–
SMTC and HMTC	3.220 (0.990–10.479)	0.052	2.595 (0.785–8.580)	0.118	0.709 (0.251–2.003)	0.516	–	–
Stage	1.100 (0.957–1.265)	0.179	–	–	1.057 (0.830–1.347)	0.654	–	–
bCt	1.000 (1.000–1.000)	0.036	1.000 (1.000–1.000)	0.316	1.000 (1.000–1.000)	0.054	1.000 (1.000–1.000)	0.711
Age	1.004 (0.984–1.024)	0.699	–	–	1.034 (1.004–1.065)	0.026	1.039 (1.008–1.071)	0.014
Gender	0.811 (0.442–1.488)	0.498	–	–	0.739 (0.390–1.400)	0.353	–	–

bCt, basal calcitonin; HMTC, hereditary MTC; MTV, metabolic tumor volume; PMTC, primary MTC; RMTC, recurrent MTC; SMTC, sporadic MTC; SUV_max_, maximum standardized uptake value; SUV_mean_, mean standardized uptake value; –, not determined.

Similarly, univariate and multivariate Cox regression analyses were performed for PMTC patients. In these analyses, age and MTV were consistently found to be significantly correlated with OS in both univariate and multivariate models. Other variables did not demonstrate predictive value for OS (Table 4).

## Analysis of the correlation of SUV_mean_ in multiple organs

SUV_mean_ for multiple organs, including the spleen, kidneys, liver, stomach, aorta, inferior vena cava, portal and splenic veins, pancreas, adrenal glands, and various lobes of the lungs, was quantified using MOOSE software.

A Cox regression analysis was conducted to elucidate the relationship between multiple organs SUV_mean_ and patient prognosis. Univariate analysis identified SUV_mean_ values of the spleen, kidneys, liver, stomach, aorta, inferior vena cava, and lung as prognostic factors for OS. Subsequent multivariate analysis refined these findings, demonstrating that SUV_mean_ of the left kidney, liver, aorta, and pancreas independently predict OS (Table 5).

**Table 5:** Univariate and multivariate Cox regression analysis of OS on the variables of organs.

Variable	Univariate analysis		Multivariate analysis	
	HR (95% CI)	*P*	HR (95% CI)	*P*
Spleen	1.614 (1.019–2.556)	0.042	0.405 (0.088–1.865)	0.246
Right kidney	1.177 (1.006–1.377)	0.042	0.769 (0.495–1.195)	0.243
Left kidney	1.340 (1.063–1.689)	0.013	2.448 (1.150–5.211)	0.020
Liver	1.719 (1.208–2.447)	0.003	3.820 (1.144–12.753)	0.029
Stomach	1.730 (1.064–2.13)	0.027	1.482 (0.578–3.796)	0.413
Aorta	1.800 (1.094–2.962)	0.021	0.057 (0.004–0.907)	0.042
Inferior vena cava	1.654 (1.043–2.624)	0.032	1.728 (0.246–12.153)	0.582
Portal vein and splenic vein	1.113 (0.755–1.639)	0.589	–	–
Pancreas	0.878 (0.690–1.118)	0.291	0.431 (0.273–0.681)	0.000
Right adrenal gland	1.111 (0.786–1.570)	0.552	–	–
Left adrenal gland	0.886 (0.593–1.323)	0.554	–	–
Left lung upper lobe	10.917 (2.406–49.539)	0.002	0.000 (0.000–19.372)	0.150
Left lung lower lobe	5.212 (1.629–16.681)	0.005	0.628 (0.003–113.319)	0.860
Right lung upper lobe	15.086 (3.302–68.918)	0.000	262.625 (0.056–1,234,668.144)	0.197
Right lung middle lobe	11.200 (2.830–44.318)	0.001	20.716 (0.033–12,828.397)	0.355
Right lung lower lobe	7.481 (2.447–22.873)	0.000	26.147 (0.063–10,891.447)	0.289

**Figure 2:**
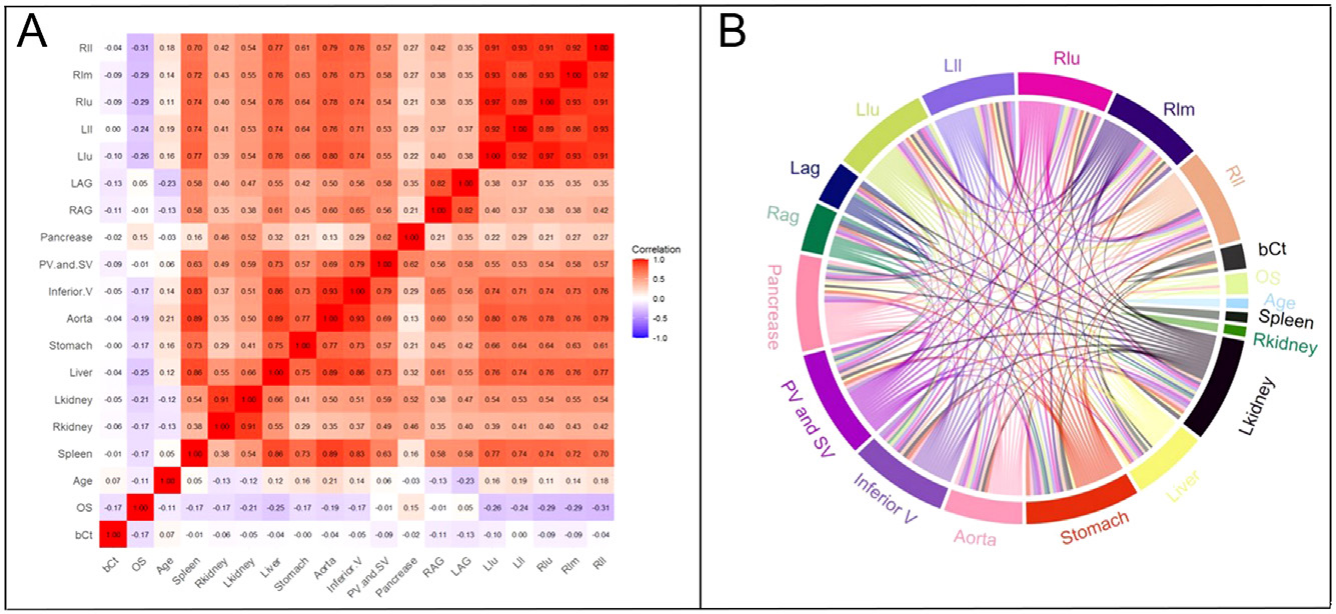
Two heat map formats to display the correlation among bCt, OS, age, and SUV_mean_ of multiple organs in all patients with MTC (A, B). The numbers in A represent the values of correlation coefficient *r*. Inferior V, inferior vena cava; LAG, left adrenal gland; Lkidney, left kidney; PV and SV, portal vein and splenic vein; RAG, right adrenal gland; Llu, upper lobe of left lung; Lll: lower lobe of left lung; PV and SV, portal vein and splenic vein; RAG, right adrenal gland; Rkidney, right kidney; Rll, lower lobe of right lung; Rlm, middle lobe of right lung; Rlu, upper lobe of right lung.

Correlations between SUV_mean_ across all assessed organs, bCt markers, OS, and patient age are presented in Fig. 2. This analysis revealed a slight negative correlation between bCt values and both OS and SUV_mean_ of the adrenal glands. Patient age exhibited a slight positive correlation with SUV_mean_ of the liver, stomach, aorta, and lungs, but a slight negative correlation with SUV_mean_ of OS, kidneys, and adrenal glands. Additionally, positive correlations of varying degrees were observed among the SUV_mean_ values of different organs.

Furthermore, a gender-based analysis was performed to investigate differences in multiple organs SUV_mean_ values. The results indicated a significantly higher uptake of [^18^F]F-DOPA PET/CT in lung tissues (with exception of right lung lower lobe) of female patients compared to male patients, while no significant differences were observed in other organs (Table 6).

**Table 6:** The difference of SUV_mean_ in organs between female and male patients.

Organ	Female	Male	*t*	*P*
Spleen	1.26 ± 0.43	1.14 ± 0.36	1.651	0.102
Right kidney	2.96 ± 1.05	2.95 ± 1.35	0.061	0.951
Left kidney	2.74 ± 0.97	2.69 ± 0.96	0.273	0.785
Liver	1.79 ± 0.61	1.79 ± 0.53	−0.015	0.988
Stomach	1.18 ± 0.46	1.09 ± 0.35	1.238	0.219
Aorta	1.20 ± 0.37	1.17 ± 0.39	0.333	0.740
Inferior vena cava	1.41 ± 0.43	1.36 ± 0.44	0.671	0.504
Portal and splenic veins	1.67 ± 0.62	1.57 ± 0.48	0.946	0.346
Pancreas	1.94 ± 1.01	1.80 ± 0.86	0.774	0.440
Right adrenal gland	1.71 ± 0.60	1.55 ± 0.64	1.282	0.203
Left adrenal gland	1.73 ± 0.59	1.55 ± 0.63	1.493	0.138
Left lung				
Upper lobe	0.38 ± 0.12	0.32 ± 0.13	2.670	0.009
Lower lobe	0.49 ± 0.16	0.42 ± 0.17	2.146	0.034
Right lung				
Upper lobe	0.38 ± 0.12	0.33 ± 0.13	2.174	0.032
Middle lobe	0.37 ± 0.13	0.30 ± 0.13	2.693	0.008
Lower lobe	0.50 ± 0.18	0.46 ± 0.17	1.277	0.205

## Discussion

To the best of our knowledge, the present study had the largest single-center cohort for investigating [^18^F]F-DOPA PET/CT in patients with MTC. Our study explored the diagnostic value of [^18^F]F-DOPA PET/CT in these patients with MTC. The patient-related sensitivity, specificity, and accuracy of [^18^F]F-DOPA PET/CT were 95%, 93%, and 94%, respectively. Our results are consistent with some previous studies (6, 8, 9, 11, 16) demonstrating the high diagnostic efficiency of [^18^F]F-DOPA PET/CT for evaluation of MTC. The patient-related sensitivity in our study is close to that recently reported by Califano *et al.* (11), but higher than those published earlier (6, 10, 17, 18, 19, 20). The differences in the patient-related sensitivity between our results and previous studies may be due to different size of cohorts and different inclusion criteria (21). In most previous studies (6, 9, 10, 19, 20, 22), the number of included patients varied from 17 to 47, and only patients undergoing restaging were included. In the present study, 109 patients were included, and we investigated both restaging patients with RMTC and staging patients with PMTC. Furthermore, true positive and true negative patients could be more accurately determined in our study due to the long follow-up period. This may partly explain the high patient-related sensitivity observed in our study. The overall lesion-related diagnostic sensitivity of [^18^F]F-DOPA PET/CT was found to be lower (65%) compared to the patient-related sensitivity (95%) and consistent with some previous studies (16, 19, 20, 22). Although [^18^F]F-DOPA PET/CT demonstrated superior performance in identifying PMTC lesions and distant metastases. The relatively lower lesion-related sensitivity was mainly due to the presence of small (<1 cm) metastases in lymph nodes and lungs, leading to false negative results. Notably, the lack of [^18^F]F-DOPA uptake in numerous small lymph node lesions resulted in a lesion-related sensitivity for lymph node metastases of only 54%. These results suggest that [^18^F]F-DOPA PET/CT may underestimate small lymph node metastasis and lung metastasis formations, warranting cautious interpretation in the assessment of MTC.

As shown in Table 1, there was a significant difference in lesion-related sensitivities of [^18^F]F-DOPA-PET/CT between PMTC (51%) and RMTC (100%) in our study. This difference might also be explained by the low sensitivity of [^18^F]F-DOPA-PET in patients with PMTC for detecting small lung lesions and small lymph node metastases in the central neck. This may be because the positive primary lesion in the thyroid on [^18^F]F-DOPA-PET may influence the visualization of small lymph node lesions in the central neck region. Our results suggested that [^18^F]F-DOPA PET/CT is better suited as a tool for advanced disease workup than for initial staging procedure.

Recently, the European Association of Nuclear Medicine (21) and European Society for Medical Oncology (23) have recommended the use of [^18^F]F-DOPA PET/CT for detecting MTC recurrences/metastases due to the high sensitivity and specificity. However, cut-off values of calcitonin for imaging with [^18^F]F-DOPA PET/CT for detection of PMTC were still controversial. In the present study, we showed the best cut-off value for imaging with [^18^F]F-DOPA in patients with bCt levels ≥64 pg/mL, with sensitivity of 89% and specificity of 73%. The 2009 American Thyroid Association (ATA) guideline (24) recommends a threshold of bCt at 150 pg/mL for performing additional imaging, including [^18^F]F-DOPA PET/CT, while Luster *et al.* (6) found that [^18^F]F-DOPA PET/CT had 100% sensitivity and specificity when bCt levels at the time of scanning were over 150 pg/mL for detecting recurrences/metastases. In our present study, the cut-off value of calcitonin is suggested also for PMTC, whereas the cut-off values of calcitonin in previous studies/guideline are recommended for detecting recurrences/metastases after primary treatment. Our present results showed the best cut-off value for imaging with [^18^F]F-DOPA in patients with RMTC ≥156 pg/mL. Therefore, our result is consistent with previous studies and guidelines. According to current ATA guideline (1) neither ^18^F-FDG PET/CT nor ^18^F-DOPA PET/CT examination was recommended to detect the presence of distant MTC metastases. However, this recommendation may be mainly based on the study results performed with [^18^F]FDG PET/CT (1, 5). Comparative studies had demonstrated that [^18^F]F-DOPA PET/CT has significantly higher sensitivity in detection of MTC lesions as compared with [^18^F]FDG PET/CT (17, 19, 20, 22).

For the diagnosis of MTC, a variety of imaging procedures can be used (25). Neck ultrasound evaluation is an important imaging method used in the diagnosis of thyroid MTC and regional neck lymph node metastases. CT is used mainly in the detection of distant metastases of MTC, whereas magnetic resonance tomography (MRT) may be very useful tool for the evaluation of liver lesions. Bone scintigraphy is a complementary and sensitive procedure to detect bone metastases. Our present results suggest that [^18^F]F-DOPA PET/CT may have an overall high sensitivity for the evaluation of MTC.

Our previous studies (3, 26, 27) have shown the gender differences in the cut-off values of plasma Ct levels in the diagnosis of MTC, as well as gender-specific differences in side effects induced by Ct stimulation tests. Similar gender-specific disparities have been reported previously (28). In this study, we showed the non-significant gender differences in sensitivity and specificity of [^18^F]F-DOPA PET/CT . We further found that the optimal bCt and sCt cut-off values for [^18^F]F-DOPA in the detection of MTC were lower in female patients as compared with those in male patients. However, there were no statistically significant differences in cut-off values between the female and male patients. Interestingly, in female patients with RMTC, the optimal bCt and sCt cut-off values were non-significantly lower than those in male patients. The results might have implication for using [^18^F]F-DOPA in RMTC patients during follow-up. The optimal bCt and sCt cut-off values for [^18^F]F-DOPA in the detection of MTC were lower in PMTC patients as compared with those in RMTC patients. Similarly, the optimal CEA cut-off values for [^18^F]F-DOPA in the detection of MTC were lower in male and PMTC patients as compared with those in female and RMTC patients. Although these differences were not statistically significant, they might have some practical implications for clinical physicians by choosing [^18^F]F-DOPA PET/CT examinations more effectively.

Our present results had also shown that there were significantly positive correlation of bCt and CEA with [^18^F]F-DOPA uptake in patients with MTC. The results were consistent with previous studies (6, 9, 10). An interesting result of our study was that a positive correlation of sCt with [^18^F]F-DOPA uptake was also shown in patients with MTC. An increase of bCt is usually associated with tumor size increase and progression of MTC (29, 30). The correlation of Ct levels and [^18^F]F-DOPA uptake could further imply that an increase of SUV_max_, SUVmean, and MTV in [^18^F]F-DOPA PET/CT examinations may be associated with progressive disease. Therefore, semi-quantitative analysis of [^18^F]F-DOPA PET/CT might play an additive role in evaluating the possible progression of MTC. Furthermore, the present study also demonstrated that tumor stage, MTC classification, and bCt levels were identified as significant predictive variables for the results of [^18^F]F-DOPA PET/CT.

In this study, the predictive variables of clinical and PET/CT parameters were analyzed in RMTC and PMTC patients. The results revealed that [^18^F]F-DOPA PET/CT positive results and high MTV were independent prognostic factors in RMTC patients and associated with poor prognosis. Meanwhile, MTC and age were independent prognostic factors in PMTC patients. Moreover, the OS in the patients of RMTC group with negative [^18^F]F-DOPA PET/CT were significantly longer than those with positive [^18^F]F-DOPA PET/CT. A similar result was recently observed by Caobelli *et al.* (31), who demonstrated the prognostic value of [^18^F]F-DOPA PET/CT in patients with RMTC. However, in contrast to the results of our present study, Caobelli *et al.* (31) found that [^18^F]F-DOPA PET parameters failed to show significant prognostic value. The differences in the prognostic value of [^18^F]F-DOPA PET parameters between our present results and those of Caobelli *et al.* may be due to the use of different PET scanners in different centers, which may have affected the measurements of [^18^F]F-DOPA PET metabolic parameters, as they already mentioned in the their study (31). Furthermore, our study had a much longer follow-up period than the previous study (31). Therefore, [^18^F]F-DOPA avidity appears to be more frequent in MTCs with poor prognosis than in those with a good prognosis. MTV may reflect the metabolic burden of tumors, which might be an important indicator of the prognosis of MTC patients.

In this study, the prognostic value of SUV_mean_ across multiple organs was evaluated in patients with MTC using [^18^F]F-DOPA PET/CT. Our Cox regression analysis revealed that SUV_mean_ values of the left kidney, liver, aorta, and pancreas are independent prognostic indicators in MTC patients. These findings suggest that SUV_mean_ measurements might serve as a preliminary tool for prognosis assessment in MTC. This approach could offer a possibility for prognostic evaluation. However, the underlying mechanisms driving these associations remain unclear and warrant further investigation. Additionally, the correlation analysis indicated a negative association between SUV_mean_ values of the spleen, kidney, liver, stomach, aorta, interior vein, and lung with OS, whereas a positive correlation was observed for the pancreas. These observations may be very interesting since this is the first report describing these specific correlations. However, further studies are necessary to validate the results.

Our study has several limitations. One of these limitations is that the number of outcome events was not large enough to separately evaluate significant OS differences within all different types of MTC. The second limitation is the limited number of patients with HMTC. MTC is a rare disease, and HMTC is even rarer. Additionally, although 109 patients were included in the present study, which constitutes one of the largest cohorts compared to previous studies, the sample size remains relatively small due to the low incidence of MTC and relatively long survival rate. Furthermore, in our present study, no comparison study between [^18^F]F-DOPA PET/CT and [^18^F]F-FDG PET/CT, as well as [^68^Ga]Ga-somatostatin receptor (SSTR) PET/CT was performed. Although [^18^F]F-DOPA is currently the best radiopharmaceutical for PET/CT in imaging MTC, additional FDG- or SSTR-PET might increase the sensitivity of PET examination, since some DOPA-negative lesions may be FDG-positive or SSTR-PET positive (32). Moreover, a positive somatostatin analog imaging opens the option of internal radiotherapy with ^177^Lu-labeled ligands of SSTR (33).

## Conclusion

[^18^F]F-DOPA PET/CT had great value for diagnosis and prognostic assessment in patients with MTC. The DOPA PET/CT parameter SUV_mean_ and MTV may have significant association to OS in Supplementary File (see section on supplementary materials given at the end of this article).

## Supplementary Materials

Supplementary Material

## Declaration of interest

ZZ is a visiting scholar at Department of Biomedical Imaging and Image-guided Therapy, Medical University of Vienna and acknowledges support of ‘The Excellent Going Abroad Experts’ Training Program in Hebei Province’. The other authors declare that there is no conflict of interest that could be perceived as prejudicing the impartiality of the study reported.

## Data availability

The datasets generated during and/or analyzed during the current study are available from the corresponding author on reasonable request.

## Ethics approval

This study was performed in line with the principles of the Declaration of Helsinki. Approval was granted by the Ethics Committee of Medical University of Vienna.

## Author contributions

Study conception and design: SL, MH; data collection and analysis: ZZ, ER, SL, JY, ZJ, GK, TT, LH; dtatistical analyses: ZZ, JY, SL, RC; manuscript writing: ZZ, SL; manuscript revision: SL. All authors have seen and approved the manuscript. ZZ and JY contributed equally to this study.
