# Supplementary material for: The role of [18F]F-DOPA PET/CT in diagnostic and prognostic assessment of medullary thyroid cancer: a 15-year experience with 109 patients
Source: Eur Thyroid J. 2024 Jul 13;13(4):e240089. doi: 10.1530/ETJ-24-0089 (PMC11301540; doi:10.1530/ETJ-24-0089)
Supplement: Supplementary Material [file supplementary_material.pdf]

The Role of [^{18}F]F-DOPA PET/CT in Diagnostic and Prognostic Assessment of Medullary Thyroid Cancer (MTC): A 15-Year Experience with 109 Patients

Zhang Z., Yu J., Rainer E., Hargitai L., Jiang Z., Karanikas G., Traub-Weidinger T., Crevenna R., Hacker M., **Li S.**

1. Methods

Participants: 109 Patients with MTC

Intervention: [^{18}F]F-DOPA PET/CT

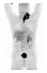

; [^{18}F]F-DOPA PET Parameter; Basal calcitonin (bCt); Stimulated calcitonin; CEA

2. Results

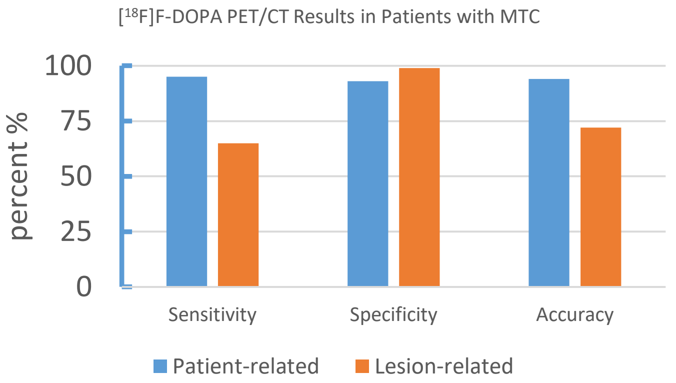

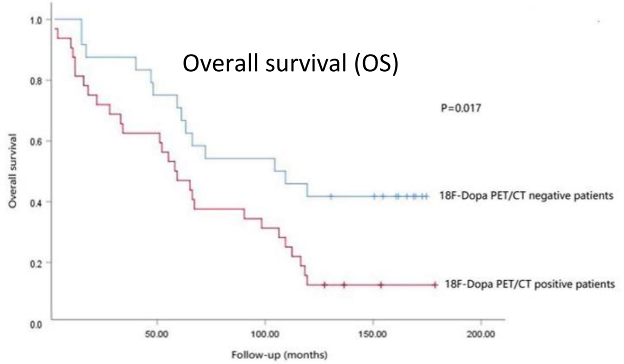

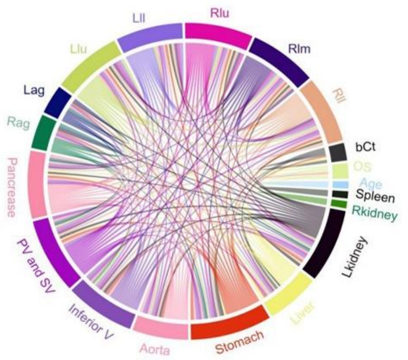

The correlation among bCt, OS, age and SUV_{mean} of multi-organs

3. Conclusions

[^{18}F]F-DOPA PET/CT had great value for diagnosis and prognostic assessment in patients with MTC.
The DOPA PET/CT parameter SUV_{mean} and MTV showed significant association to Overall Survival (OS)
